# *rac*-(4*R*,17*S*,18*R*,26*R*)-Ethyl 4′-methoxy­carbonyl-5′′-(4-methoxy­phen­yl)-1′-methyl-2,3′′-dioxo-2′′,3′′-dihydro­indoline-3-spiro-2′-pyrrolidine-3′-spiro-2′′-thia­zolo[3,2-*a*]pyrimidine-6′′-carboxyl­ate

**DOI:** 10.1107/S160053680900261X

**Published:** 2009-01-28

**Authors:** Zhao-Hui Hou, Ning-Bo Zhou, Bin-Hong He, Xiao-Fang Li

**Affiliations:** a Department of Chemistry and Chemical Engineering, Hunan Institute of Science and Technology, Yueyang 414000, People’s Republic of China; b School of Chemistry and Chemical Engineering, Hunan University of Science and Technology, Xiangtan 411201, People’s Republic of China

## Abstract

In the title compound, C_30_H_30_N_4_O_7_S, the two spiro junctions link a planar 2-oxindole ring [with a mean deviation from the plane of 0.0319 (3) Å, a pyrrolidine ring in an envelope conformation and a thia­zolo[3,2-*a*]pyrimidine system. Two mol­ecules are connected into a dimer by two N—H⋯O hydrogen bonds, forming an *R*_2_^2^(8) graph-set motif. The title compound has four stereogenic centers and appears as a racemic mixture of one single diastereoisomer (*RSRR*/*SRSS*).

## Related literature

For related literature on spiro compounds, see: Caramella & Grunanger (1984 ▶); James *et al.* (1991 ▶); Kobayashi *et al.* (1991 ▶). For structural discussion, see: Cremer & Pople (1975 ▶); Etter (1990 ▶); Bernstein *et al.* (1994 ▶).
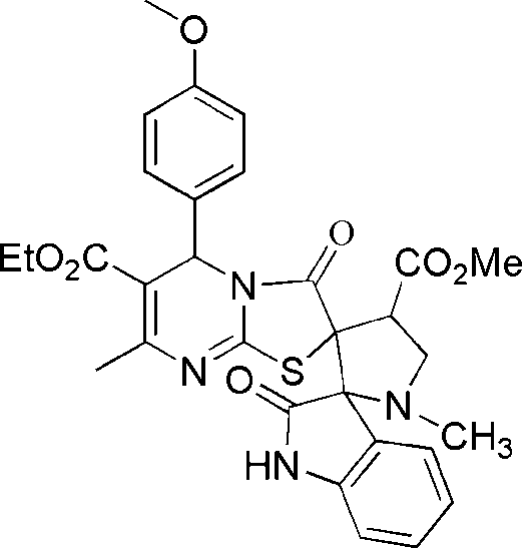


## Experimental

### 

#### Crystal data

C_30_H_30_N_4_O_7_S
*M* *_r_* = 590.64
Triclinic, 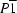

*a* = 9.944 (2) Å
*b* = 11.389 (2) Å
*c* = 13.417 (3) Å
α = 98.06 (3)°
β = 107.36 (3)°
γ = 101.00 (3)°
*V* = 1391.8 (6) Å^3^
*Z* = 2
Mo *K*α radiation
μ = 0.17 mm^−1^
*T* = 113 (2) K
0.20 × 0.18 × 0.08 mm

#### Data collection

Rigaku Saturn diffractometer
Absorption correction: multi-scan (*CrystalClear*; Rigaku, 2001 ▶) *T*_min_ = 0.956, *T*_max_ = 0.977
10220 measured reflections
4881 independent reflections
3700 reflections with *I* > 2σ(*I*)
*R*_int_ = 0.029

#### Refinement

*R*[*F* ^2^ > 2σ(*F* ^2^)] = 0.035
*wR*(*F* ^2^) = 0.098
*S* = 1.06
4881 reflections
384 parameters
H-atom parameters constrained
Δρ_max_ = 0.28 e Å^−3^
Δρ_min_ = −0.22 e Å^−3^

### 

Data collection: *CrystalClear* (Rigaku, 2001 ▶); cell refinement: *CrystalClear*; data reduction: *CrystalClear*; program(s) used to solve structure: *SHELXS97* (Sheldrick, 2008 ▶); program(s) used to refine structure: *SHELXL97* (Sheldrick, 2008 ▶); molecular graphics: *SHELXTL* (Sheldrick, 2008 ▶) and *PLATON* (Spek, 2003 ▶); software used to prepare material for publication: *SHELXTL*.

## Supplementary Material

Crystal structure: contains datablocks I, global. DOI: 10.1107/S160053680900261X/dn2427sup1.cif

Structure factors: contains datablocks I. DOI: 10.1107/S160053680900261X/dn2427Isup2.hkl

Additional supplementary materials:  crystallographic information; 3D view; checkCIF report

## Figures and Tables

**Table 1:** Hydrogen-bond geometry (Å, °)

*D*—H⋯*A*	*D*—H	H⋯*A*	*D*⋯*A*	*D*—H⋯*A*
N3—H3⋯O5^i^	0.86	1.97	2.8189 (18)	169

Symmetry code: (i) 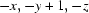.

**Fig. 1.**
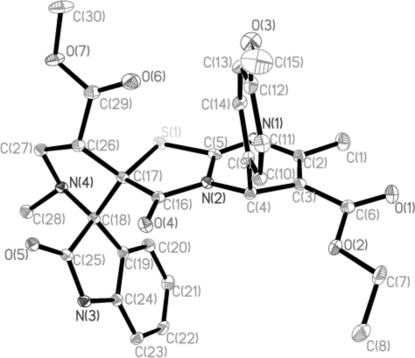
The molecular structure of (I) with the atom-labeling scheme. Ellipsoids are drawn at the 30% probability level. H atoms have been omitted for clarity.

**Fig. 2.**
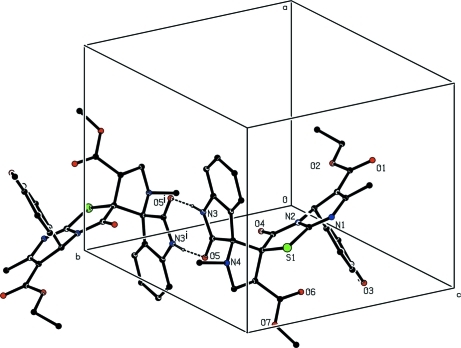
Partial packing view showing the formation of dimer through N-H···O hydrogen bonds. H atoms not involved in hydrogen bondings have been omitted for clarity. [Symmetry code: (i) -x, 1-y, -z]

## supplementary crystallographic information

### Comment

Spiro-compounds represent an important class of naturally occurring substances, which in many cases exhibit important biological properties (Kobayashi *et al.*, 1991; James *et al.*, 1991). 1,3-Dipolar cycloaddition reactions are widely used for the construction of spiro-compounds (Caramella & Grunanger,1984). In this paper, the structure of the title compound (I) is reported. The compound was synthesized by the intermolecular [3 + 2] cycloaddition of azomethine ylide, derived from isatin and sarcosine by a decarboxylative route, and (2*Z*)-ethyl 2-((methoxycarbonyl)methylene)-3,5-dihydro-5-(4-methoxyphenyl)- 7-methyl-3-oxo-2*H*-thiazolo[3,2-*a*]pyrimidine-6-carboxylate.

In the title compound, C_30_H_30_N_4_O_7_S, the two spiro junctions link a planar 2-oxindole ring, a pyrrolidine ring in an half-chair conformation and a thiazolo[3,2-*a*]pyrimidine ring (Fig. 1). The pyrrolidine ring (N4/C27/C26/C17/C18) has a half-chair conformation with puckering parameters, Q(2)= 0.4780 (18)Å and φ(2)= 47.9° (Cremer & Pople, 1975). The 2-oxindole ring (N3/C25/C18/C19/C20/C21/C22/C23/C24) is nearly planar with the mean deviation from this plane being 0.032 (3)%A.

Two molecules are connected into a dimer by two N—H···O hydrogen bonds forming a ring with a R_2_^2^(8) graph set motif (Etter, 1990; Bernstein *et al.*, 1994) (Table 1, Fig. 2).

The title compound has 4 stereogenic centers and then appears as a racemic mixture of one single diastereoisomer (*RSRR*/*SRSS*).

### Experimental

A mixture of (2*Z*)-ethyl 2-((methoxycarbonyl)methylene)-3,5-dihydro-5-(4-methoxyphenyl)- 7-methyl-3-oxo-2*H*-thiazolo[3,2-*a*]pyrimidine-6-carboxylate(1 mmol), isatin(1 mmol) and sarcosine(1 mmol) were refluxed in methanol (60 ml) until the disappearence of the starting material as evidenced by the TLC. After the reaction was over, the solvent was removed *in vacuo* and the residue was separated by column chromatography (silica gel, petroleum ether/ethylacetate=5:1) to give the title compound (I).

m.p.497 K; ^1^H-NMR (δ, p.p.m.): 1.01–1.02(m, 3H), 2.06 (s, 3H), 2.35(s, 3H), 3.05(s, 3H), 3.39–3.40 (m, 1H), 3.60–3.63 (m, 1H), 3.90–3.92(m, 2H), 4.81–4.85(m, 1H), 5.76(s, 1H), 6.74–6.76(m, 1H), 6.96–6.99(m, 1H), 7.20–7.26(m, 5H), 7.58–7.60(m, 1H), 7.62 (bs, 1H, –NH);

20 mg of (I) was dissolved in 15 ml dioxane-ethyl acetate mixed solvent; the solution was kept at room temperature for 15 d by natural evaporation to give colorless single crystals of (I), suitable for X-Ray analysis.

### Refinement

All H atoms attached to C atoms and N atom were fixed geometrically and treated as riding with C—H = 0.96Å (methyl), 0.97Å (methylene), 0.98Å (methine) and N—H = 0.86 Å with U_iso_(H) = 1.2U_eq_(C or N) or U_iso_(H) = 1.5U_eq_(methine).

### Crystal data

**Table:**

C_30_H_30_N_4_O_7_S	*Z* = 2
*M**_r_* = 590.64	*F*(000) = 620
Triclinic, *P*1	*D*_x_ = 1.409 Mg m^−^^3^
Hall symbol: -P 1	Mo *K*α radiation, λ = 0.71073 Å
*a* = 9.944 (2) Å	Cell parameters from 4258 reflections
*b* = 11.389 (2) Å	θ = 1.8–27.9°
*c* = 13.417 (3) Å	µ = 0.17 mm^−^^1^
α = 98.06 (3)°	*T* = 113 K
β = 107.36 (3)°	Block, colourless
γ = 101.00 (3)°	0.20 × 0.18 × 0.08 mm
*V* = 1391.8 (6) Å^3^	

### Data collection

**Table:**

Rigaku Saturn diffractometer	4881 independent reflections
Radiation source: rotating anode	3700 reflections with *I* > 2σ(*I*)
confocal	*R*_int_ = 0.029
ω scans	θ_max_ = 25.0°, θ_min_ = 1.9°
Absorption correction: multi-scan (*CrystalClear*; Rigaku, 2001)	*h* = −11→11
*T*_min_ = 0.956, *T*_max_ = 0.977	*k* = −13→10
10220 measured reflections	*l* = −15→15

### Refinement

**Table:**

Refinement on *F*^2^	Primary atom site location: structure-invariant direct methods
Least-squares matrix: full	Secondary atom site location: difference Fourier map
*R*[*F*^2^ > 2σ(*F*^2^)] = 0.035	Hydrogen site location: inferred from neighbouring sites
*wR*(*F*^2^) = 0.098	H-atom parameters constrained
*S* = 1.06	*w* = 1/[σ^2^(*F*_o_^2^) + (0.0589*P*)^2^] where *P* = (*F*_o_^2^ + 2*F*_c_^2^)/3
4881 reflections	(Δ/σ)_max_ < 0.001
384 parameters	Δρ_max_ = 0.28 e Å^−^^3^
0 restraints	Δρ_min_ = −0.22 e Å^−^^3^

### Special details

**Table:**

Geometry. All esds (except the esd in the dihedral angle between two l.s. planes) are estimated using the full covariance matrix. The cell esds are taken into account individually in the estimation of esds in distances, angles and torsion angles; correlations between esds in cell parameters are only used when they are defined by crystal symmetry. An approximate (isotropic) treatment of cell esds is used for estimating esds involving l.s. planes.
Refinement. Refinement of *F*^2^ against ALL reflections. The weighted *R*-factor *wR* and goodness of fit *S* are based on *F*^2^, conventional *R*-factors *R* are based on *F*, with *F* set to zero for negative *F*^2^. The threshold expression of *F*^2^ > σ(*F*^2^) is used only for calculating *R*-factors(gt) *etc*. and is not relevant to the choice of reflections for refinement. *R*-factors based on *F*^2^ are statistically about twice as large as those based on *F*, and *R*- factors based on ALL data will be even larger.

### Fractional atomic coordinates and isotropic or equivalent isotropic displacement parameters (Å^2^)

**Table:**

	*x*	*y*	*z*	*U*_iso_*/*U*_eq_	
S1	0.04156 (5)	0.35302 (4)	0.42322 (3)	0.02231 (13)	
O1	0.32486 (14)	−0.13252 (12)	0.32935 (11)	0.0354 (3)	
O2	0.26663 (13)	−0.05624 (11)	0.18117 (10)	0.0281 (3)	
O3	−0.42217 (12)	−0.30677 (11)	0.05644 (10)	0.0316 (3)	
O4	−0.02319 (12)	0.24227 (10)	0.12268 (9)	0.0236 (3)	
O5	−0.11369 (12)	0.48453 (10)	0.08582 (8)	0.0218 (3)	
O6	−0.27264 (14)	0.17208 (12)	0.27772 (13)	0.0429 (4)	
O7	−0.39268 (13)	0.31585 (11)	0.30007 (10)	0.0328 (3)	
N1	0.16597 (15)	0.16869 (13)	0.46129 (11)	0.0241 (3)	
N2	0.07144 (14)	0.18849 (12)	0.28126 (10)	0.0174 (3)	
N3	0.13510 (14)	0.52812 (12)	0.12989 (11)	0.0208 (3)	
H3	0.1406	0.5309	0.0674	0.025*	
N4	−0.02410 (14)	0.56993 (12)	0.32782 (10)	0.0182 (3)	
C1	0.3131 (2)	0.02959 (19)	0.51724 (15)	0.0344 (5)	
H1A	0.3593	−0.0288	0.4913	0.052*	
H1B	0.2512	−0.0083	0.5526	0.052*	
H1C	0.3858	0.0978	0.5667	0.052*	
C2	0.22450 (18)	0.07314 (16)	0.42587 (14)	0.0229 (4)	
C3	0.20286 (17)	0.03199 (15)	0.32123 (14)	0.0213 (4)	
C4	0.09652 (17)	0.07312 (14)	0.23379 (13)	0.0189 (4)	
H4	0.1403	0.0886	0.1790	0.023*	
C5	0.10029 (17)	0.22161 (15)	0.39038 (13)	0.0199 (4)	
C6	0.27164 (17)	−0.06142 (16)	0.28186 (15)	0.0239 (4)	
C7	0.3202 (2)	−0.14574 (17)	0.12533 (16)	0.0364 (5)	
H7A	0.2439	−0.2201	0.0913	0.044*	
H7B	0.4011	−0.1660	0.1752	0.044*	
C8	0.3682 (2)	−0.09030 (19)	0.04354 (17)	0.0391 (5)	
H8A	0.2880	−0.0685	−0.0041	0.059*	
H8B	0.4014	−0.1483	0.0037	0.059*	
H8C	0.4459	−0.0184	0.0783	0.059*	
C9	−0.04382 (17)	−0.02596 (14)	0.18261 (13)	0.0181 (4)	
C10	−0.07297 (18)	−0.10076 (15)	0.08378 (13)	0.0212 (4)	
H10	−0.0079	−0.0867	0.0468	0.025*	
C11	−0.19697 (18)	−0.19597 (15)	0.03911 (14)	0.0250 (4)	
H11	−0.2141	−0.2460	−0.0267	0.030*	
C12	−0.29533 (17)	−0.21597 (15)	0.09342 (14)	0.0232 (4)	
C13	−0.26702 (18)	−0.14307 (16)	0.19270 (14)	0.0248 (4)	
H13	−0.3321	−0.1574	0.2297	0.030*	
C14	−0.14238 (17)	−0.04920 (15)	0.23686 (13)	0.0216 (4)	
H14	−0.1240	−0.0009	0.3037	0.026*	
C15	−0.4605 (2)	−0.3746 (2)	−0.04918 (17)	0.0525 (6)	
H15A	−0.4643	−0.3196	−0.0975	0.079*	
H15B	−0.5540	−0.4305	−0.0685	0.079*	
H15C	−0.3891	−0.4195	−0.0531	0.079*	
C16	0.00520 (17)	0.26045 (14)	0.21851 (13)	0.0182 (4)	
C17	−0.02451 (17)	0.36688 (15)	0.28420 (12)	0.0179 (4)	
C18	0.04721 (17)	0.49640 (14)	0.27189 (12)	0.0177 (4)	
C19	0.21177 (17)	0.53663 (14)	0.31052 (13)	0.0190 (4)	
C20	0.31437 (18)	0.56616 (16)	0.41158 (14)	0.0244 (4)	
H20	0.2864	0.5586	0.4711	0.029*	
C21	0.46058 (18)	0.60752 (16)	0.42303 (15)	0.0275 (4)	
H21	0.5304	0.6264	0.4907	0.033*	
C22	0.50340 (18)	0.62089 (16)	0.33501 (15)	0.0262 (4)	
H22	0.6016	0.6480	0.3443	0.031*	
C23	0.40141 (17)	0.59426 (15)	0.23323 (14)	0.0227 (4)	
H23	0.4291	0.6039	0.1739	0.027*	
C24	0.25706 (17)	0.55281 (14)	0.22340 (13)	0.0194 (4)	
C25	0.00866 (18)	0.49965 (14)	0.14971 (13)	0.0187 (4)	
C26	−0.18551 (17)	0.37201 (15)	0.25039 (13)	0.0197 (4)	
H26	−0.2205	0.3585	0.1723	0.024*	
C27	−0.17881 (17)	0.50704 (15)	0.29251 (13)	0.0208 (4)	
H27A	−0.2352	0.5409	0.2364	0.025*	
H27B	−0.2161	0.5145	0.3516	0.025*	
C28	0.00420 (19)	0.69933 (15)	0.32462 (14)	0.0244 (4)	
H28A	0.1071	0.7349	0.3524	0.037*	
H28B	−0.0424	0.7401	0.3672	0.037*	
H28C	−0.0334	0.7083	0.2522	0.037*	
C29	−0.28448 (18)	0.27477 (16)	0.27959 (14)	0.0257 (4)	
C30	−0.5040 (2)	0.22464 (19)	0.31518 (18)	0.0452 (6)	
H30A	−0.5414	0.1575	0.2545	0.068*	
H30B	−0.5813	0.2604	0.3225	0.068*	
H30C	−0.4628	0.1956	0.3785	0.068*	

### Atomic displacement parameters (Å^2^)

**Table:**

	*U*^11^	*U*^22^	*U*^33^	*U*^12^	*U*^13^	*U*^23^
S1	0.0323 (3)	0.0206 (2)	0.0145 (2)	0.00589 (18)	0.00909 (19)	0.00318 (18)
O1	0.0359 (8)	0.0347 (8)	0.0425 (8)	0.0188 (6)	0.0128 (7)	0.0170 (7)
O2	0.0311 (7)	0.0284 (7)	0.0330 (7)	0.0159 (6)	0.0164 (6)	0.0083 (6)
O3	0.0246 (7)	0.0246 (7)	0.0345 (7)	−0.0027 (5)	0.0041 (6)	−0.0046 (6)
O4	0.0335 (7)	0.0232 (6)	0.0143 (6)	0.0093 (5)	0.0074 (5)	0.0024 (5)
O5	0.0213 (6)	0.0286 (7)	0.0150 (6)	0.0065 (5)	0.0048 (5)	0.0057 (5)
O6	0.0429 (8)	0.0239 (7)	0.0705 (11)	0.0051 (6)	0.0322 (8)	0.0122 (7)
O7	0.0267 (7)	0.0302 (7)	0.0435 (8)	−0.0003 (6)	0.0213 (7)	0.0026 (6)
N1	0.0255 (8)	0.0268 (8)	0.0190 (7)	0.0050 (6)	0.0058 (7)	0.0065 (7)
N2	0.0198 (7)	0.0160 (7)	0.0159 (7)	0.0036 (6)	0.0062 (6)	0.0025 (6)
N3	0.0227 (8)	0.0270 (8)	0.0153 (7)	0.0064 (6)	0.0090 (6)	0.0064 (6)
N4	0.0219 (7)	0.0164 (7)	0.0177 (7)	0.0047 (6)	0.0094 (6)	0.0019 (6)
C1	0.0336 (11)	0.0395 (11)	0.0282 (10)	0.0130 (9)	0.0036 (9)	0.0103 (9)
C2	0.0182 (9)	0.0241 (9)	0.0269 (9)	0.0038 (7)	0.0070 (8)	0.0108 (8)
C3	0.0148 (8)	0.0214 (9)	0.0276 (9)	0.0025 (7)	0.0066 (8)	0.0091 (8)
C4	0.0197 (9)	0.0181 (8)	0.0197 (8)	0.0055 (7)	0.0083 (7)	0.0019 (7)
C5	0.0212 (9)	0.0204 (9)	0.0164 (8)	0.0005 (7)	0.0066 (7)	0.0043 (7)
C6	0.0141 (9)	0.0219 (9)	0.0326 (10)	−0.0006 (7)	0.0061 (8)	0.0065 (8)
C7	0.0389 (12)	0.0299 (10)	0.0495 (13)	0.0199 (9)	0.0216 (11)	0.0070 (10)
C8	0.0419 (12)	0.0425 (12)	0.0411 (12)	0.0189 (10)	0.0223 (11)	0.0054 (10)
C9	0.0190 (9)	0.0166 (8)	0.0194 (8)	0.0070 (7)	0.0053 (7)	0.0045 (7)
C10	0.0240 (9)	0.0220 (9)	0.0203 (8)	0.0079 (7)	0.0092 (8)	0.0053 (7)
C11	0.0307 (10)	0.0217 (9)	0.0188 (9)	0.0069 (8)	0.0049 (8)	−0.0011 (8)
C12	0.0183 (9)	0.0182 (9)	0.0275 (9)	0.0042 (7)	0.0014 (8)	0.0012 (8)
C13	0.0218 (9)	0.0248 (9)	0.0295 (10)	0.0056 (7)	0.0130 (8)	0.0012 (8)
C14	0.0224 (9)	0.0192 (9)	0.0208 (9)	0.0042 (7)	0.0072 (8)	−0.0020 (7)
C15	0.0482 (14)	0.0483 (14)	0.0340 (12)	−0.0169 (11)	0.0018 (11)	−0.0114 (11)
C16	0.0169 (8)	0.0167 (8)	0.0190 (9)	0.0003 (7)	0.0063 (7)	0.0020 (7)
C17	0.0217 (9)	0.0179 (8)	0.0125 (8)	0.0025 (7)	0.0057 (7)	0.0016 (7)
C18	0.0218 (9)	0.0172 (8)	0.0138 (8)	0.0043 (7)	0.0063 (7)	0.0025 (7)
C19	0.0215 (9)	0.0155 (8)	0.0205 (9)	0.0056 (7)	0.0074 (8)	0.0034 (7)
C20	0.0283 (10)	0.0243 (9)	0.0200 (9)	0.0068 (8)	0.0069 (8)	0.0053 (8)
C21	0.0210 (9)	0.0296 (10)	0.0254 (9)	0.0060 (8)	−0.0005 (8)	0.0031 (8)
C22	0.0181 (9)	0.0233 (9)	0.0364 (10)	0.0067 (7)	0.0083 (8)	0.0037 (8)
C23	0.0231 (9)	0.0210 (9)	0.0261 (9)	0.0065 (7)	0.0117 (8)	0.0025 (8)
C24	0.0232 (9)	0.0163 (8)	0.0189 (8)	0.0063 (7)	0.0069 (7)	0.0027 (7)
C25	0.0258 (10)	0.0146 (8)	0.0165 (8)	0.0046 (7)	0.0088 (8)	0.0025 (7)
C26	0.0198 (9)	0.0202 (9)	0.0185 (8)	0.0029 (7)	0.0075 (7)	0.0019 (7)
C27	0.0230 (9)	0.0222 (9)	0.0186 (8)	0.0062 (7)	0.0091 (8)	0.0032 (7)
C28	0.0300 (10)	0.0185 (9)	0.0255 (9)	0.0059 (7)	0.0106 (8)	0.0048 (8)
C29	0.0270 (10)	0.0255 (10)	0.0241 (9)	0.0035 (8)	0.0114 (8)	0.0012 (8)
C30	0.0389 (12)	0.0398 (12)	0.0564 (14)	−0.0091 (10)	0.0317 (12)	−0.0001 (11)

### Geometric parameters (Å, °)

**Table:**

S1—C5	1.7542 (17)	C9—C14	1.393 (2)
S1—C17	1.8239 (17)	C10—C11	1.386 (2)
O1—C6	1.204 (2)	C10—H10	0.9300
O2—C6	1.347 (2)	C11—C12	1.388 (2)
O2—C7	1.4547 (19)	C11—H11	0.9300
O3—C12	1.375 (2)	C12—C13	1.386 (2)
O3—C15	1.417 (2)	C13—C14	1.382 (2)
O4—C16	1.2092 (19)	C13—H13	0.9300
O5—C25	1.225 (2)	C14—H14	0.9300
O6—C29	1.195 (2)	C15—H15A	0.9600
O7—C29	1.336 (2)	C15—H15B	0.9600
O7—C30	1.449 (2)	C15—H15C	0.9600
N1—C5	1.279 (2)	C16—C17	1.525 (2)
N1—C2	1.418 (2)	C17—C26	1.543 (2)
N2—C16	1.372 (2)	C17—C18	1.565 (2)
N2—C5	1.386 (2)	C18—C19	1.516 (2)
N2—C4	1.4763 (19)	C18—C25	1.576 (2)
N3—C25	1.351 (2)	C19—C20	1.381 (2)
N3—C24	1.410 (2)	C19—C24	1.395 (2)
N3—H3	0.8600	C20—C21	1.394 (2)
N4—C28	1.456 (2)	C20—H20	0.9300
N4—C18	1.4613 (19)	C21—C22	1.387 (2)
N4—C27	1.468 (2)	C21—H21	0.9300
C1—C2	1.489 (3)	C22—C23	1.387 (2)
C1—H1A	0.9600	C22—H22	0.9300
C1—H1B	0.9600	C23—C24	1.383 (2)
C1—H1C	0.9600	C23—H23	0.9300
C2—C3	1.354 (2)	C26—C29	1.511 (2)
C3—C6	1.488 (2)	C26—C27	1.544 (2)
C3—C4	1.522 (2)	C26—H26	0.9800
C4—C9	1.517 (2)	C27—H27A	0.9700
C4—H4	0.9800	C27—H27B	0.9700
C7—C8	1.490 (3)	C28—H28A	0.9600
C7—H7A	0.9700	C28—H28B	0.9600
C7—H7B	0.9700	C28—H28C	0.9600
C8—H8A	0.9600	C30—H30A	0.9600
C8—H8B	0.9600	C30—H30B	0.9600
C8—H8C	0.9600	C30—H30C	0.9600
C9—C10	1.388 (2)		
			
C5—S1—C17	92.62 (8)	O3—C15—H15B	109.5
C6—O2—C7	118.23 (14)	H15A—C15—H15B	109.5
C12—O3—C15	116.59 (14)	O3—C15—H15C	109.5
C29—O7—C30	115.34 (15)	H15A—C15—H15C	109.5
C5—N1—C2	116.85 (14)	H15B—C15—H15C	109.5
C16—N2—C5	117.35 (13)	O4—C16—N2	123.53 (14)
C16—N2—C4	121.33 (13)	O4—C16—C17	124.42 (15)
C5—N2—C4	120.99 (14)	N2—C16—C17	112.04 (13)
C25—N3—C24	112.18 (13)	C16—C17—C26	114.29 (13)
C25—N3—H3	123.9	C16—C17—C18	114.60 (12)
C24—N3—H3	123.9	C26—C17—C18	100.14 (13)
C28—N4—C18	116.07 (12)	C16—C17—S1	106.54 (11)
C28—N4—C27	114.43 (13)	C26—C17—S1	110.98 (11)
C18—N4—C27	107.91 (12)	C18—C17—S1	110.29 (11)
C2—C1—H1A	109.5	N4—C18—C19	115.48 (13)
C2—C1—H1B	109.5	N4—C18—C17	99.08 (11)
H1A—C1—H1B	109.5	C19—C18—C17	117.85 (14)
C2—C1—H1C	109.5	N4—C18—C25	113.92 (13)
H1A—C1—H1C	109.5	C19—C18—C25	101.49 (12)
H1B—C1—H1C	109.5	C17—C18—C25	109.54 (13)
C3—C2—N1	122.34 (16)	C20—C19—C24	119.01 (15)
C3—C2—C1	126.15 (16)	C20—C19—C18	131.99 (14)
N1—C2—C1	111.49 (15)	C24—C19—C18	108.80 (14)
C2—C3—C6	123.73 (17)	C19—C20—C21	118.98 (16)
C2—C3—C4	121.49 (15)	C19—C20—H20	120.5
C6—C3—C4	114.66 (14)	C21—C20—H20	120.5
N2—C4—C9	111.87 (12)	C22—C21—C20	121.00 (17)
N2—C4—C3	108.35 (13)	C22—C21—H21	119.5
C9—C4—C3	110.37 (13)	C20—C21—H21	119.5
N2—C4—H4	108.7	C23—C22—C21	120.73 (15)
C9—C4—H4	108.7	C23—C22—H22	119.6
C3—C4—H4	108.7	C21—C22—H22	119.6
N1—C5—N2	126.15 (15)	C24—C23—C22	117.44 (15)
N1—C5—S1	122.36 (13)	C24—C23—H23	121.3
N2—C5—S1	111.45 (13)	C22—C23—H23	121.3
O1—C6—O2	123.06 (16)	C23—C24—C19	122.81 (16)
O1—C6—C3	127.42 (17)	C23—C24—N3	127.24 (15)
O2—C6—C3	109.52 (15)	C19—C24—N3	109.84 (14)
O2—C7—C8	107.49 (15)	O5—C25—N3	126.46 (15)
O2—C7—H7A	110.2	O5—C25—C18	125.94 (13)
C8—C7—H7A	110.2	N3—C25—C18	107.53 (14)
O2—C7—H7B	110.2	C29—C26—C17	115.01 (14)
C8—C7—H7B	110.2	C29—C26—C27	117.66 (13)
H7A—C7—H7B	108.5	C17—C26—C27	103.79 (13)
C7—C8—H8A	109.5	C29—C26—H26	106.5
C7—C8—H8B	109.5	C17—C26—H26	106.5
H8A—C8—H8B	109.5	C27—C26—H26	106.5
C7—C8—H8C	109.5	N4—C27—C26	104.93 (13)
H8A—C8—H8C	109.5	N4—C27—H27A	110.8
H8B—C8—H8C	109.5	C26—C27—H27A	110.8
C10—C9—C14	118.29 (14)	N4—C27—H27B	110.8
C10—C9—C4	120.96 (13)	C26—C27—H27B	110.8
C14—C9—C4	120.62 (14)	H27A—C27—H27B	108.8
C11—C10—C9	121.39 (15)	N4—C28—H28A	109.5
C11—C10—H10	119.3	N4—C28—H28B	109.5
C9—C10—H10	119.3	H28A—C28—H28B	109.5
C10—C11—C12	119.42 (15)	N4—C28—H28C	109.5
C10—C11—H11	120.3	H28A—C28—H28C	109.5
C12—C11—H11	120.3	H28B—C28—H28C	109.5
O3—C12—C13	115.67 (15)	O6—C29—O7	124.42 (16)
O3—C12—C11	124.42 (15)	O6—C29—C26	124.14 (15)
C13—C12—C11	119.90 (15)	O7—C29—C26	111.27 (15)
C14—C13—C12	120.14 (15)	O7—C30—H30A	109.5
C14—C13—H13	119.9	O7—C30—H30B	109.5
C12—C13—H13	119.9	H30A—C30—H30B	109.5
C13—C14—C9	120.84 (15)	O7—C30—H30C	109.5
C13—C14—H14	119.6	H30A—C30—H30C	109.5
C9—C14—H14	119.6	H30B—C30—H30C	109.5
O3—C15—H15A	109.5		

### Hydrogen-bond geometry (Å, °)

**Table:**

*D*—H···*A*	*D*—H	H···*A*	*D*···*A*	*D*—H···*A*
N3—H3···O5^i^	0.86	1.97	2.8189 (18)	169

Symmetry codes: (i) −*x*, −*y*+1, −*z*.
